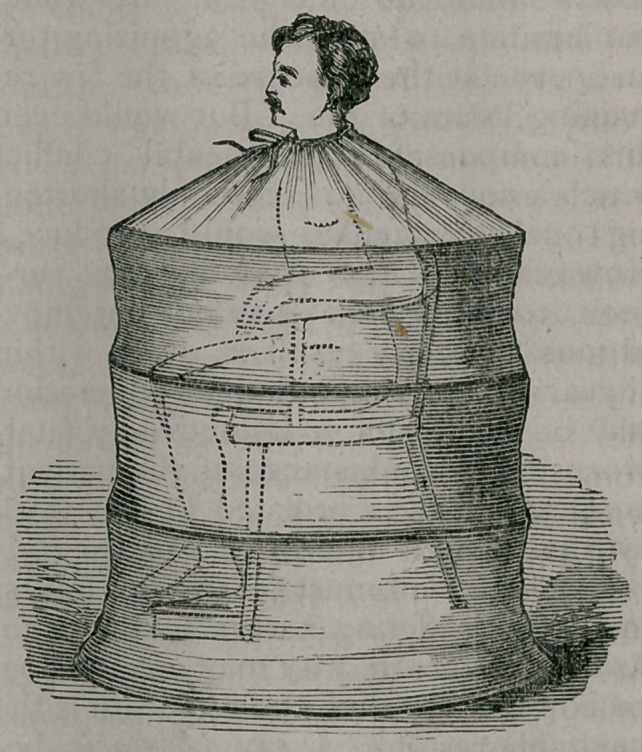# Fumigation in Syphilis

**Published:** 1877-12-20

**Authors:** 


### FUMIGATION IN SYPHILIS.

Prof. D. W. Yandell, University of Louisville, in a clinical lecture, thus describes his method of using the *mercurial vapor bath*:

I show you a sketch of the chair, stand, and gas burners we have used for many years past.

A less expensive, though it is also a much less durable heater than this, is that which I hold in my hands. The burning fluid in this is alcohol. It consists, you will observe, of a sheet-iron stand, which contains two lamps and two dishes, one capable of containing a pint of water, the other, which is quite shallow, being intended for the mercury.

A very convenient apparatus is that of my friend, Dr. Frank Maury, of Philadelphia, which is intended, as you will remark, for gas, but to which alcohol lamps may also be adapted.

I next show you the cloak, made in this instance of canton flannel, and supported and made to stand away from the body of the patient by four hoops. It fastens, reticule-fashion, well around the neck, and has, you will see, a slit in front through which, when it is desired, some of the vapor may be allowed to escape. The chair, the cloak, the lamp, the pans, thus constitute the apparatus.

But there is yet a cheaper and still a simpler rig than this, consisting of a large blanket, a hot brick, and a bucket of boiling water. The blanket must have a slit in the center, and be slipped over the head, Mexican fashion. The brick should be hot enough to vaporize the calomel. The water in the bucket should be boiling, that it may furnish the needed steam.

I have seen this homely apparatus accomplish many brilliant cures. Then it is found at hand in every family, occupies no space, excites no inquiry among other members of a household, and, better, than all, may be used by the patient himself without assistance. Among the humbler classes, and especially where secrecy is imperative, you will find this means of fumigation to serve you a really excellent purpose.

And now a word as to the way in which the bath is given. See to it that the room in which you intend to work is well warmed. Nothing, I think, in the whole process will reward you better than attention to this very point. Pour, in the summer season, half a pint of water into the pan; in the winter let it be a pint. Bring this to a boil. Put on the plate twenty to forty or sixty grains of calomel. Have your patient strip, seat himself in the chair, and cover with the wrap. Draw it well up about his neck, and have it come down full to the floor. In a few minutes the steam from the water will produce a pleasant sense of warmth and moisture. Now light the burner under the calomel. So arrange the flame that it will require fifteen to twenty minutes to consume the mercury. Should the heat at any time grow excessive, lower the jet under the water, or extinguish it altogether. About every five minutes open the slit in the wrap at the patient's throat, and have him breathe the vapors for a minute or so. If this produces coughing, stop it: the calomel is probably volatilized too rapidly, or is not as pure as it should be. At the end of fifteen or twenty minutes shut off the light under the water. If the calomel be vaporized, which it usually is in this time, stop that jet also; otherwise wait a few moments. The detention will not be for long, when you may put out the light, and, after giving the patient time to cool, have him remove the wrap, and, in ordinary cases, dress himself. Should you desire to bring him under the dominion of the mercury with the least loss of time, have him, instead, put on a long canton flannel or woolen shirt and get into bed; and when there give him a tumblerful of compound decoction of guiacum or sarsaparilla, and give it hot; and thus you will prolong the action of his skin which the bath has started.

At another time I may tell you of these and other allies which, in need, you may summon to your aid. For the present, however, you must content yourselves with a lesson in the mere mechanical part of the work, volatized.

And though I have dwelt upon this at such length, I must still add that you will not always be able to give the baths just in the manner I have described. Your own knowledge of disease will teach you this. Circumstances alter cases in our business as much as they do in other callings; and there is a long bill of exceptions to the general rules I have been endeavoring to lay down for your guidance. Among them I may mention that your patient will sometimes grow faint, and hurry you to shutting off the heat. Sometimes, again, he will be slow to sweat, and you will have to raise your fires. Again, he will be so ready to perspire—to run all away to sweat—that you will be forced to use but a few spoonsful of water, and but barely enough heat under the mercury to vaporize it. At another time he will, through some carelessness of yours, or of his, get more of the fumes of the calomel than either his bronchi or his stomach will bear, and he will have a coughing spell, or a nausea, which will oblige you to suspend operations. In yet another instance, he may be so exhausted by disease as to make a full bath quite beyond his strength, and here you will have to touch the heat, and the water, and the mercury, all very lightly.

These matters, which are so important in themselves, and which will influence in so large a degree both the satisfaction and the success with which you will use the baths, I can now only hint at. Had I time to multiply details, even to wearying you, the ultimate fact would still remain, that, in giving the baths, you should be constantly on the alert lest, on the one hand, you oppress the patient, or on the other allow the enemy which you are assailing to check your advance.

Mr. Parker employed from a pint to a quart of water at a bath, while Mr. Lee thinks an ounce sufficient. Mr. Parker employed two lamps; Mr. Lee employed but one. For my part, I am quite clear that the amount of steam and the volatilization of the mercury can be somewhat better regulated with two burners than with one; hence you see me use two.

In the last years of Mr. Parker's life he practically abandoned all other preparations of mercury for the bisulphuret, and for calomel, and employed them either alone or together. Mr. Lee, who was the first to use calomel in this way, remains true to his early preference, and has never, I think, employed anything else. Calomel is, as you see, also my stand-by; I prefer it to any other preparation. In a few rare and excessively rebellious cases of syphilis, assailing especially the skin and cellular tissue, I have thought I got better results by putting the bisulphuret and calomel together—in the proportion of one drachm of the former to half a drachm of the latter—than I did from either preparation used singly. Mr. Parker believed that the bisulphuret possessed a special power over the rupial form of syphilis, and I confess to sharing this belief with that lamented surgeon.

But whichever preparation you select, take the trouble to test its purity. Remember my first experience with the bisulphuret. And in the matter of calomel, too, though it may be of the best brand, you can render its fumes still less irritating to the air-passages by resubliming and then washing it. You will hear, in another room, how these processes rid the calomel of its free hydrochloric acid, the vapors of which vex the lungs not only of him who takes, but also of him who gives, the bath. I myself can not breathe the fumes of other than the purest calomel without being almost suffocated by cough, and made exceedingly wretched generally.

Mr. Lee thinks it of so much importance to have the contact of the calomel with the surface of the body maintained for a considerable period of time, that he lays exceeding stress on the patient, after the bath, getting into bed with the cloak or wrap on, and using it as a night dress. Mr. Parker, on the contrary, attached no weight to this view, but directed his patients to be rubbed dry on leaving the bath.

Now, both these surgeons have recorded exceptional success in the management of syphilis, though they have obtained it by methods which differ no little in what would seem to be quite important particulars. This I think should teach you that the essence of the treatment fortunately lies deeper than the mere externals by which it is achieved. And it should also convince you that the aggregate of morbid actions which constitutes syphilis may be reached very certainly, and counteracted very thoroughly, by the same means applied in very different ways. Just, for instance, as any of you may get from this city to New York by rail on many different routes. And you will do so in much the same time and way, though the agents and friends of rival lines may tell you quite another story. When you have traveled on them all, as I have done, you come to find that each line of the road, as each line of treatment, has advantages and drawbacks quite peculiar to itself. What one route saves you in time, it may cost you in comfort; while the comfort afforded by a third may be at the expense of your safety.

I am very sure Mr. Parker's method is a good one, because he has so declared it, and I can point to hundreds of cases which attest it. I am very sure that Mr. Lee's method is a good one, because Mr. Lee says so, and I can point you to hundreds of cases, all of which go to prove it. Yet, after saying this, I must add that I follow neither method exclusively. To be plain, gentlemen, it isn't every man with the pock who is so situated that, after his bath, he can draw this not very picturesque drapery about him, and lie upon his couch till morning. Most bathers must dress and go about their business as soon as they are through with their sweat. I am convinced, that it is a serious hindrance to the cure. I wish, indeed, that all my syphilitics, and, as for the matter of that, all of yours, too —when you have them—belonged to the richer classes, and could afford the time and the money to jump from the bath into bed. But such is not the case; most must run while they bathe.

What, then, are you to do under these circumstances ?

Simply this: —Have your man well cooled before he is uncovered, and he will thus need no special drying. Should his surface, however, remain overmoist or he fancy a rubbing, let him have it. No material harm will come of the little operation. It may perhaps, delay in a slight degree, the effect of the bath, but the cure will none the less go on. The mercury may not tell on the disease nor on the gums altogether as soon, but it will tell just as surely as though the patient had gone to bed wrapped in his cloak. I do not think I can be mistaken in this. In confirmation of it, however, let me add that one of my colleagues, Dr. L. P. Yandell, Jr.—who has superintended the administration of the baths in several thousand cases—is clearly of the opinion that brisk friction, after the sweat, made with the coarsest towel, and until the skin is all aglow, actually promotes the action of the mercury, and conduces, he thinks, to its more rapid absorption by the surface.

Now, concerning the differences on this point—and they are really very wide—you must pardon me for saying that, in my opinion, they have been invested with an importance quite beyond their actual deserts.

The best time for giving the baths is at night, just before retiring. I am positive as to this. When such an hour can not be chosen, take that which comes midway between meals. And whatever else you do, never sweat your patient on a full stomach.

And now I have done. But I beg you in conclusion, to avoid the conceit of believing that, because you may have learned how to give mercurial fumigations, you also know how to treat syphilis. There is a long list of other matters—matters of diet, and of dress, and of things far weightier than these—which you must master before you can be considered fit to undertake the management of this many-sided disease.—*Amer. Prac.*

## Figures and Tables

**Figure f1:**
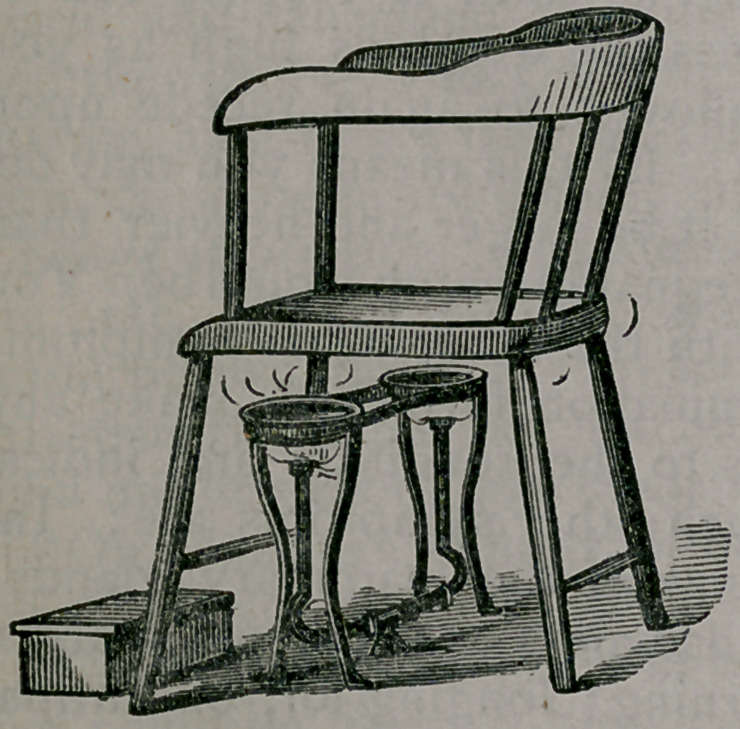


**Figure f2:**
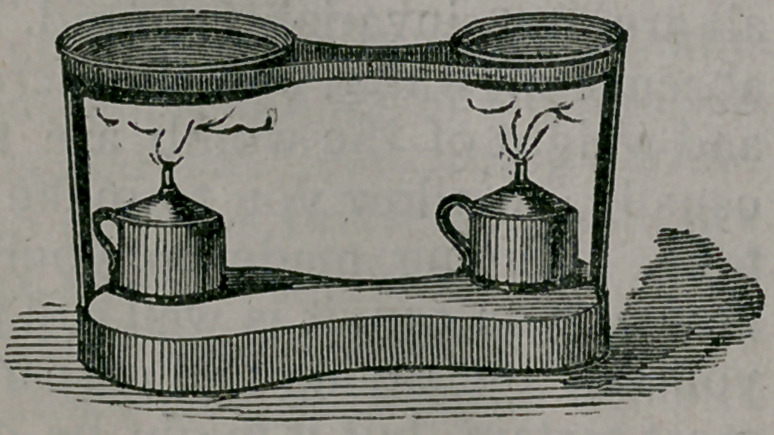


**Figure f3:**
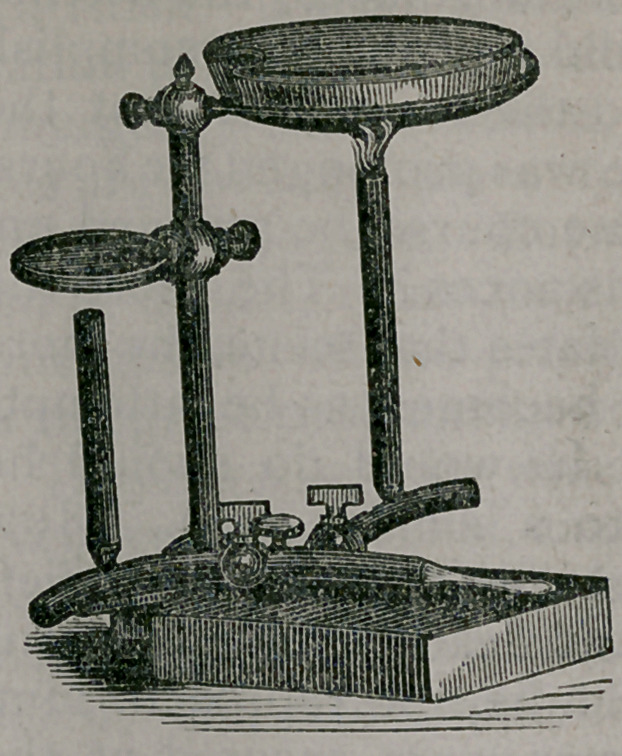


**Figure f4:**